# Tetrabenzononacene: “Butterfly Wings” Stabilize the Core

**DOI:** 10.1002/anie.201909614

**Published:** 2019-12-17

**Authors:** Matthias Müller, Steffen Maier, Olena Tverskoy, Frank Rominger, Jan Freudenberg, Uwe H. F. Bunz

**Affiliations:** ^1^ Organisch-Chemisches Institut Ruprecht-Karls-Universität Heidelberg Im Neuenheimer Feld 270 69120 Heidelberg Germany; ^2^ Centre for Advanced Materials Im Neuenheimer Feld 225 69120 Heidelberg Germany; ^3^ InnovationLab Speyerer Straße 4 69115 Heidelberg Germany

**Keywords:** acenes, benzannulation, Cava reaction, nonacene, X-ray diffraction

## Abstract

In combination with bulky substituents at the core, fourfold benzannulation at the *cata*‐positions stabilizes a nonacene sufficiently to allow its isolation and characterization by ^1^H NMR and X‐ray analysis. The four benzo units blueshift the absorption spectrum in comparison to a solely linear nonacene, but significantly increase the stability in the solid state.

## Graphical abstract

**Attachment of four benzo units** electronically stabilizes a nonacene core without heteroatom substitution at/on the aromatic backbone. The four benzo units blueshift the absorption spectrum in comparison to a linear nonacene and significantly increase the stability in the solid state.

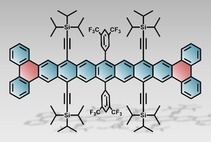

The higher acenes have been a magnificent challenge, accepted since Clar's first synthesis of pentacene.^1^ Unsubstituted, they are both insoluble and vulnerable towards ambient conditions. Anthony et al.^2^ have introduced bulky silylethynyl substituents to the larger acenes, and, depending on size and steric demand, even heptacenes can be stabilized (Figure 1).^3^ However, the stabilization of octacenes and nonacenes remains challenging. Apart from surface‐4 or matrix‐based^5^ approaches, to the best of our knowledge only two approaches yielded nonacene‐type structures. Miller et al. employed thioether substituents,^6^ while Anthony et al. combined steric repulsion with fluorination^7^ to achieve the stabilization of nonacene derivative **Non**. Other methods of stabilization allow a significant number of linearly annulated benzene rings but with starkly diminished acene character.^8^ We recently prepared stable tetrabenzoheptacenes (and azaheptacenes) such as **B_4_Hep**, exhibiting hexacene‐like absorption maxima, the blueshift being a consequence of the diminished conjugation of the quadruply annulated acene core.^9^ Here we extend this approach to a reasonably stable nonacene derivative, **B_4_Non**, by employing a modification of Anthony's route.

**Figure 1:**
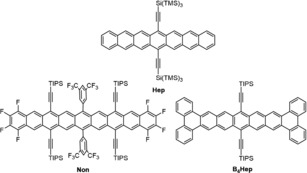
Heptacene **Hep**, nonacene **Non** and tetrabenzoheptacene **B_4_Hep**.

Bis(bromomethyl)phenanthrene **1** and an excess of *p*‐benzoquinone furnished **2** (Scheme 1); a second Cava reaction with **3** results in **4** (crude yield 87 %), fourfold ethynylation of which gave intermediate **5** (40 %) using a large excess of lithium acetylide (≈100 equiv). Reductive aromatization with SnCl_2_ furnishes tetrabenzononacene **B_4_Non**. The concentration was adjusted to precipitate **B_4_Non** during synthesis. **B_4_Non**, unlike other higher acenes,^7^ is surprisingly stable in the solid state. Its proton NMR spectrum shows sharp resonances (see the Supporting Information), in contrast to the broad signals observed for other nonacenes^7^—the well‐resolved signals being due to the stabilization of its closed‐shell ground state.

**Figure 2:**
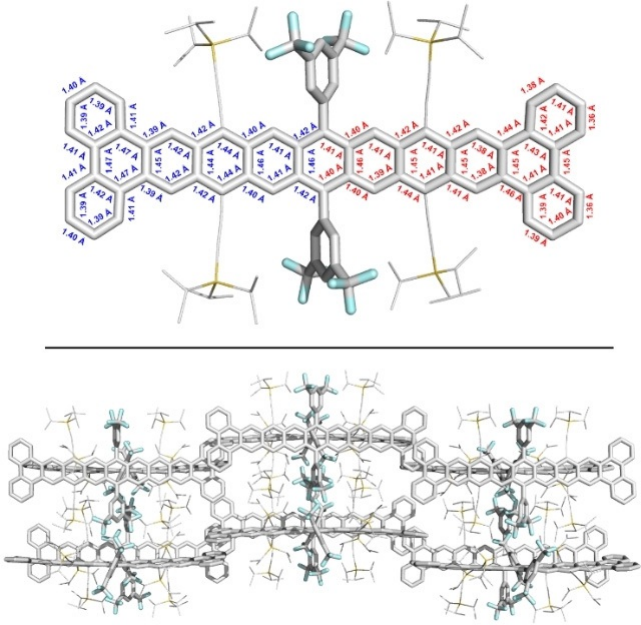
X‐ray structure (top) and packing motif (bottom) of **B_4_Non** including average bond lengths determined by crystal analysis (red) vs. calculated bond lengths (blue; DFT, B3LYP/6‐311+G*). For the sake of clarity, all protons were omitted and TIPS‐ethynyl substituents were reduced in size.

**Scheme 1:**
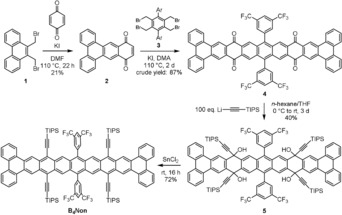
Synthesis of tetrabenzononacene **B_4_Non**.

Single crystals were grown by subsequently layering *n*‐hexane and MeOH on a THF solution of **B_4_Non** under nitrogen; solvent molecules are included in the crystal packing but are heavily disordered and cannot be resolved. **B_4_Non** crystallizes with two independent molecules per unit cell with a minor π–π interaction of two phenanthrenylenes. These molecules form 1D‐stacks and are oriented perpendicular to each other, effectively preventing dimerization but also pronounced π–π interactions in the solid state. The independent molecules’ average bond lengths of the innermost ring of the formal triphenylene units are elongated (1.44–1.47 Å) compared to that of the other aromatic C−C backbone bonds (1.38–1.46 Å).^9^ Calculated and experimentally determined bond lengths of **B_4_Non** are in agreement with each other and the expected values.

Non‐fluorescent **B_4_Non** exhibits structured, finger‐like absorption bands with a lowest energy absorption maximum at 958 nm (Figure 3, Table 1), 576 cm^−1^ blue‐shifted in comparison to that of Anthony's nonacene **Non** and red‐shifted by 2859 cm^−1^ compared to the p‐band of **B_4_Hep**. The first reduction potential of **B_4_Non** occurs at −1.19 V (cyclovoltammetry, reversible, Table 1, SI), more negative than that of **Non** (−0.51 eV) due to the absence of electron‐withdrawing fluorine substituents.^7^, ^9^ The first oxidation potential is at 0.98 V (irreversible).

**Figure 3:**
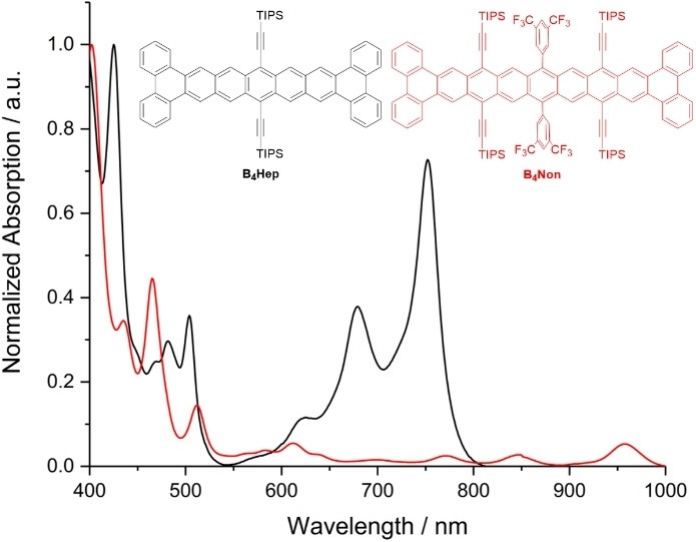
UV/vis absorption spectra of **B_4_Hep** (black) and **B_4_Non** (red) in *n*‐hexane (*c*≈10^−6^ m) at room temperature.

**Table 1:** Photophysical and calculated properties.

Acene	*λ*_max,abs_ [nm]^[a]^	*E*_1/2_^red1^ [V]^[b]^	*EA*_CV_ [eV]^[c]^	*E*_LUMO,DFT_ [eV]^[d]^
**B_4_Non**	958	−1.19	−3.61	−3.79
**Non** 7	1014	−0.51	−4.29	−4.27
**B_4_Hep** 9	752	−1.34	−3.46	−3.26
**Hep** 3	835	−0.83*	−3.97	−3.38

[a] Lowest energy absorption maxima. [b] First reduction potentials measured by cyclic voltammetry (CV) in CH_2_Cl_2_ using Bu_4_NPF_6_ as electrolyte and Fc/Fc^+^ as internal standard (−4.80 eV) at 0.2 V s^−1/^ * vs. SCE.^11^ [c] Electron affinities estimated from first reduction potentials. [d] DFT‐calculated LUMOs using TURBOMOLE B3LYP/ def2 TZVP//Gaussian 09, B3LYP/6‐311++G**. TMS substituents were used instead of TIPS to simplify calculations.^12^

Similar to most higher acenes,3a, 7, 10 there is a dramatic difference between solid state and solution persistability: **B_4_Non** is stable for more than 6 weeks under nitrogen in the solid state, although its half‐life in *n*‐hexane solution is only 30 min under ambient conditions (see SI) and 7 h under nitrogen atmosphere (Figure 4). In air probably the *endo*‐peroxide forms (see SI),^7^ while the mode of decomposition under nitrogen is less clear.

**Figure 4:**
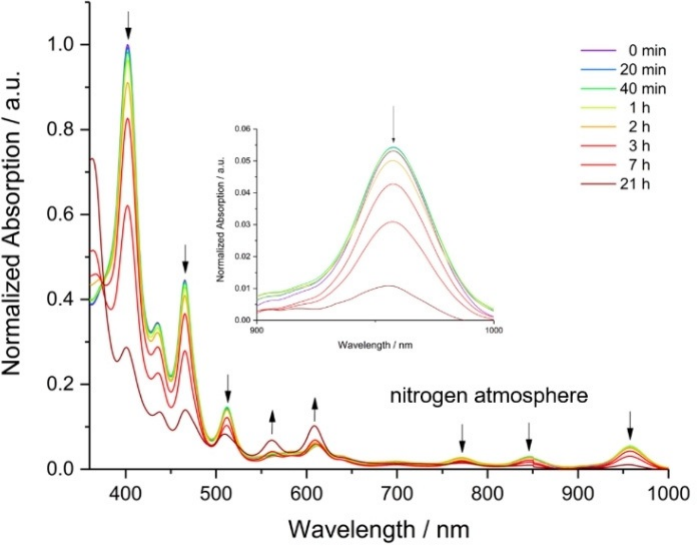
Change in UV/vis absorption intensity of **B_4_Non** under an atmosphere of nitrogen in *n*‐hexane at room temperature.

Analysis of the decomposition products via recycling gel permeation chromatography (see SI) suggests the formation of dimeric and oligomeric species, both common degradation products for higher acenes.3a, 10 Compared to **Non**, **B_4_Non** is less stable under air and nitrogen in solution (Figure 4), as **Non's** electron‐withdrawing halogen substituents retard *endo*‐peroxide formation. In contrast, **Non** is persistent for 2 d at 10 °C in the solid state,^7^ while **B_4_Non** is stable for weeks.

Figure 5 displays the calculated NICS values for **B_4_Non′**. The system shows NICS(1) values in accord with expectations, with the formal inner triphenylene ring being the least aromatic one—“empty” in the Clar formalism—and the other rings displaying high aromaticity. This is further illustrated in FMO calculations (see SI), in which the central triphenylene rings show small coefficients in the outer rings compared to their nonacene congeners.

**Figure 5:**
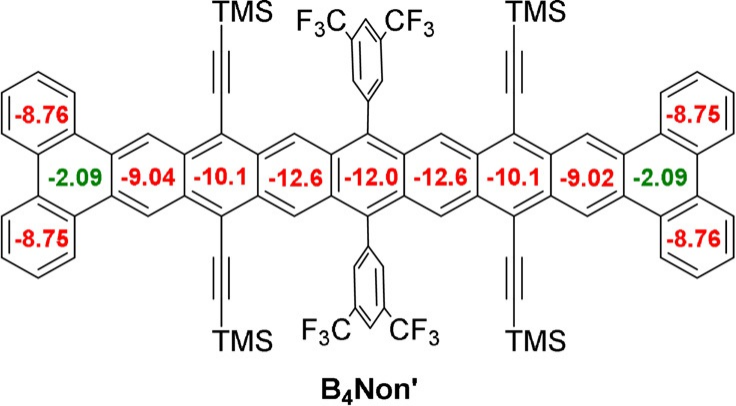
NICS(1) values of **B_4_Non′** DFT‐calculated at the B3LYP/6–311+G* level; TMS substituents were used to simplify calculations.^12^

In conclusion, we have prepared a novel, reasonably stable tetrabenzononacene, **B_4_Non**, in which the stabilization is due to the attachment of four benzo units at the *cata*‐position of the acene unit. The material displays sharp NMR resonances and a slightly blue‐shifted absorption in comparison to Anthony's nonacene **Non**, yet it is only one of the very few isolated and structurally characterized nonacenes. **B_4_Non** is highly stable in the solid state. Benzo‐wings therefore should allow stabilization of other, hitherto only moderately persistent, reactive aromatics.

## Experimental Section

CCDC https://www.ccdc.cam.ac.uk/services/structures?id=doi:10.1002/anie.201909614 (**B_4_Non**) contains the supplementary crystallographic data for this publication. These data can be obtained free of charge from The http://www.ccdc.cam.ac.uk/. The synthetic details for precursors and remaining materials can be found in the SI.

Synthesis of **6**: (Tri‐*iso*‐propylsilyl)acetylene (1.10 g, 1.36 mL, 6.02 mmol, 100 equiv.) was dissolved in *n*‐hexane (20 mL) and cooled to 0 °C. *n*‐BuLi (2.5 m in *n*‐hexane, 2.29 mL, 5.72 mmol, 95.0 equiv.) was added dropwise and the mixture was stirred for 1 h at room temperature. **4** (70.0 mg, 60.2 μmol, 1.00 equiv.) was added and the suspension was stirred for 3 d at room temperature. Sat. NHCl_4_ solution and DCM were added, the layers were separated and the aqueous layer was extracted with DCM. The combined organic layers were dried over MgSO_4_, filtered and the solvent was removed under reduced pressure. The crude product was subjected to column chromatography (SiO_2_, PE/DCM 80:20 to 25:75) to yield **6** as a yellow powder (46.0 mg, 24.3 μmol, 40 %). Mp: >350 °C. ^1^H NMR (600 MHz, CD_2_Cl_2_) *δ*=9.41 (s, 4 H), 8.80 (dd, *J=*7.2, 1.8 Hz, 4 H), 8.70 (m, 4 H), 8.47 (s, 4 H), 8.29 (d, *J=*2.0 Hz, 2 H), 8.18 (d, *J=*1.6 Hz, 4 H), 7.71 (qd, *J=*7.2, 1.6 Hz, 8 H), 3.43 (s, 4 H), 1.08 (m, 42 H), 0.97 ppm (m, 84 H). ^13^C{^1^H} NMR (151 MHz, CD_2_Cl_2_) *δ*=140.7, 137.7, 136.8, 135.8, 132.5, 132.3, 130.5, 130.4, 130.3, 129.5, 128.1, 127.6, 124.6, 124.6, 124.1, 123.6, 122.8, 122.0, 109.4, 108.9, 104.6, 90.3, 85.7, 69.4, 18.5, 18.5, 18.5, 11.4, 11.3 ppm. IR (neat): *ν* (cm^−1^)=2942, 2863, 1464, 1335, 1274, 1141, 1042, 888, 750, 670. HRMS (MALDI^+^, DCTB): *m*/*z* calcd for C_114_H_116_F_12_O_4_Si_4_: [*M*+H]^+^ 1891.7988, found: 1891.7974, correct isotope distribution.

Synthesis of **B_4_Non**: **6** (20.0 mg, 10.6 μmol, 1.00 equiv.) was dissolved in MeCN/THF (1:1, 2 mL) in a glove box. Anhydrous SnCl_2_ (40.1 mg, 211 μmol, 20.0 equiv.) was added and the mixture was stirred at room temperature overnight. The resulting precipitate was filtered, washed with MeCN and dried to give almost pure **B_4_Non** (crude yield 72 %). The material can further be purified by washing it thoroughly with DCM to yield pure **B_4_Non** (total yield: 3.0 mg, 1.64 μmol, 16 %). Suitable specimen for single crystal analysis were grown by subsequently layering *n*‐hexane and MeOH on a THF solution under nitrogen. Mp: >350 °C. ^1^H NMR (600 MHz, [D_8_]THF) *δ*=10.83 (s, 2 H), 9.72 (s, 4 H), 8.95 (s, 4 H), 8.76 (d, *J=*8.0 Hz, 4 H), 8.61 (d, *J=*8.0 Hz, 4 H), 8.49 (m, 4 H), 7.64 (m, 8 H), 1.26 ppm (m, 84 H). The compound was not soluble enough for ^13^C{^1^H} NMR analysis. IR (neat): *ν* (cm^−1^)=2939, 2855, 1453, 1369, 1213, 1110, 1019, 882, 753, 506. HRMS (MALDI^+^, DCTB): *m*/*z* calcd for C_114_H_105_F_12_Si_4_: [*M*+H]^+^ 1823.7879, found: 1823.7891, correct isotope distribution.

## Conflict of interest

The authors declare no conflict of interest.

## Supporting information

As a service to our authors and readers, this journal provides supporting information supplied by the authors. Such materials are peer reviewed and may be re‐organized for online delivery, but are not copy‐edited or typeset. Technical support issues arising from supporting information (other than missing files) should be addressed to the authors.

Supplementary
