# Supplementary material for: Tetrabenzononacene: “Butterfly Wings” Stabilize the Core
Source: Angew Chem Int Ed Engl. 2019 Dec 17;59(5):1966–9. doi: 10.1002/anie.201909614 (PMC7004125; doi:10.1002/anie.201909614)
Supplement: Supplementary file 1 — Supplementary [file ANIE-59-1966-s001.pdf]

Angewandte Chemie
Eine Zeitschrift der Gesellschaft Deutscher Chemiker

Supporting Information

Tetrabenzononacene: “Butterfly Wings” Stabilize the Core

*Matthias Müller, Steffen Maier, Olena Tverskoy, Frank Rominger, Jan Freudenberg, and Uwe H. F. Bunz**

anie_201909614_sm_miscellaneous_information.pdf

Supporting Information

Table of Contents

1 Experimental Procedures 3
1.1 Materials and Methods 3
1.2 Synthesis 3
2 Results and Discussion 4
2.1 Calculations 4
2.2 NMR Spectroscopy 5
2.3 IR Spectroscopy 7
2.4 UV/vis Absorption Stability Study 9
2.5 Cyclic Voltammetry 10
2.6 Crystallographic Data 11
2.7 Analysis of Decomposition Products 12
References 16
Author Contributions 16

1 Experimental Procedures

1.1 Materials and Methods

Column chromatography was performed using silica gel from Macherey, Nagel & Co. (particle size: 0.032−0.062 mm). NMR spectra were recorded on Bruker Avance Spectrometers using the specified frequency. Chemical shifts (δ) are given in parts per million (ppm) relative to internal solvent signals.[S1] The following abbreviations describe the signal multiplicities: s = singlet, d = doublet, dd = doublet of doublets, m = multiplet. High-resolution mass spectra (HRMS) were obtained by (matrix-assisted) laser desorption/ionization (LDI/MALDI) using *trans*-2-[3-(4-*tert*-butylphenyl)-2-methyl-2-propenylidene]malononitrile (DCTB) as matrix, electrospray ionisation (ESI) or direct analysis in real time (DART) experiments. IR Spectra were recorded from neat oil or powder of the respective analyte on a Jasco FT/IR-4100 spectrometer. CV measurements were performed on a VersaSTAT 3 potentiostat by Princeton Applied Research. UV-vis spectra were recorded on a Jasco V670. Computational studies were carried out using DFT calculations on Turbomole 6.3.1 or Gaussian 09. Geometry optimizations were performed using the B3LYP functional and def2-TZVP basis set. At this geometry, the absolute energy and FMO energies were assigned by a single-point approach at the B3LYP/6–311++G** level.[S2] 9,10-Bis(bromomethyl)phenanthrene (**1**)[S3] and 2',3',5',6'-tetrakis(bromomethyl)-3,3'',5,5''-tetrakis(trifluoromethyl)-1,1':4',1''-terpheny (**3**)l[S4] were synthesized according to literature procedures.

1.2 Synthesis

Bis(bis(trifluoromethyl)phenyl)tetrabenzononacene-tetraquinone (4)

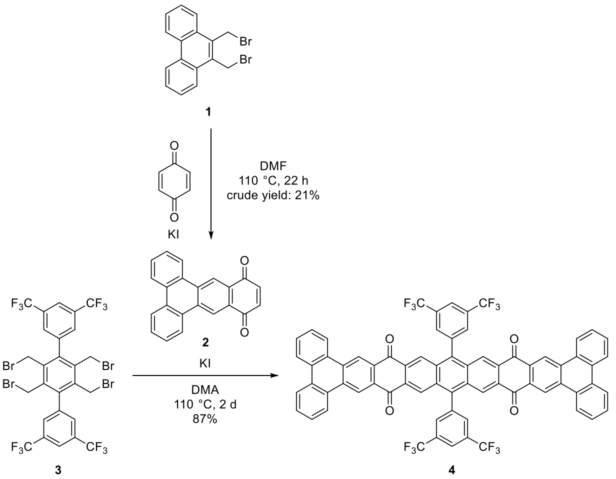

9,10-Bis(bromomethyl)phenanthrene (**1**, 4.20 g, 11.5 mmol, 1.00 eq.), *p*-benzoquinone (18.7 g, 173 mmol, 15.0 eq.) and KI (19.2 g, 115 mmol, 10.0 eq.) were dissolved in dry DMF (50 mL) under Ar. The mixture was stirred at 110 °C for 22 h and allowed to cool to room temperature. The yellow precipitate was filtered through a sinter funnel, washed with water, MeOH and acetone to yield benzo[*f*]tetraphene-10,13-dione (**2**, 760 mg, 2.46 mmol, 21%) as a yellow powder, which was directly used in the next step without further purification due to limited solubility. 2',3',5',6'-Tetrakis(bromomethyl)-3,3'',5,5''-tetrakis(trifluoromethyl)-1,1':4',1''-terphenyl (110 mg, 126 µmol, 1.00 eq.) and **2** (99.0 mg, 320 µmol, 2.55 eq.) were dissolved in dry dimethylacetamide (DMA, 8 mL) and heated to 110 °C. KI (293 mg, 1.76 mmol, 14.0 eq.) was added at once and the mixture stirred at 110 °C for 2 d. The hot reaction mixture was poured into water (120 mL) and the resulting precipitate was filtered through a sinter funnel, washed with water, acetone, THF and *n*-pentane to yield **4** (128 mg, 110 µmol, 87%) as a yellow, insoluble powder. Mp: > 350 °C. The compound was not soluble enough for NMR analysis. IR (neat): ν (cm^{-1}) = 2361, 1680, 1601, 1357, 1274, 1190, 1130, 1034, 901, 742. MS (LDI$^+$): *m/z* calcd. for $C_{70}H_{31}F_{12}O_4$: [M+H]$^+$ 1163.203, found: 1163.985, correct isotope distribution.

2 Results and Discussion

2.1 Calculations

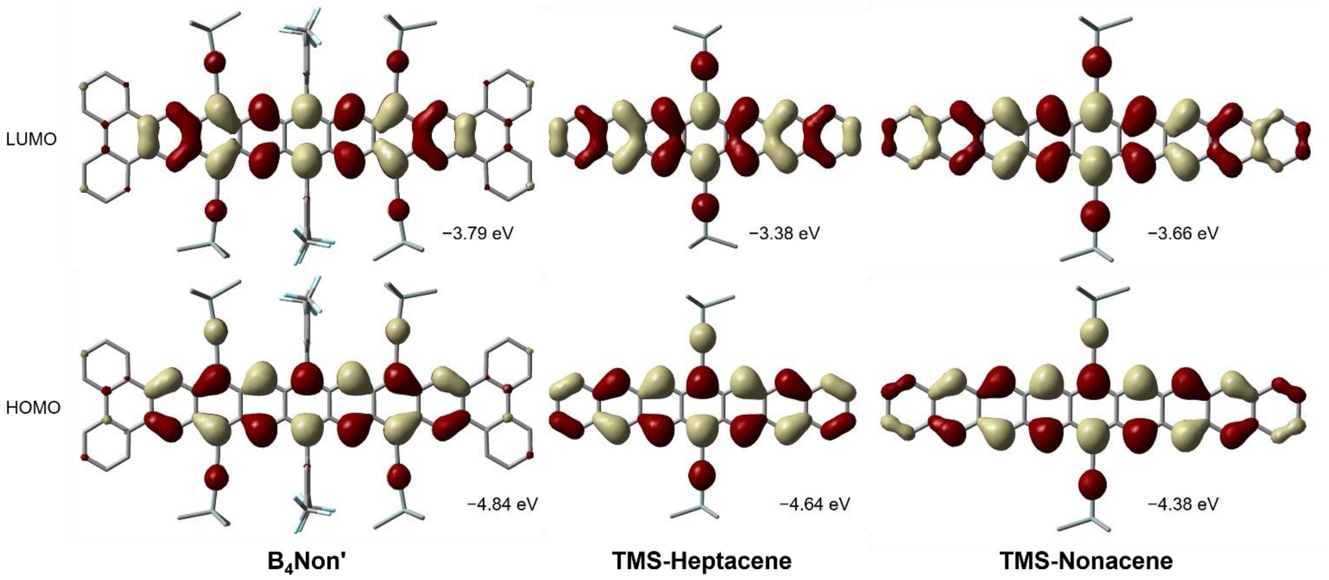

Figure S1. FMO distribution of **B_4Non'**, **TMS-Heptacene** and **TMS-Nonacene** calculated using TURBOMOLE B3LYP/def2 TZVP// B3LYP/6-311++G**. TMS groups were used instead of TIPS groups to simplify calculations.

2.2 NMR Spectroscopy

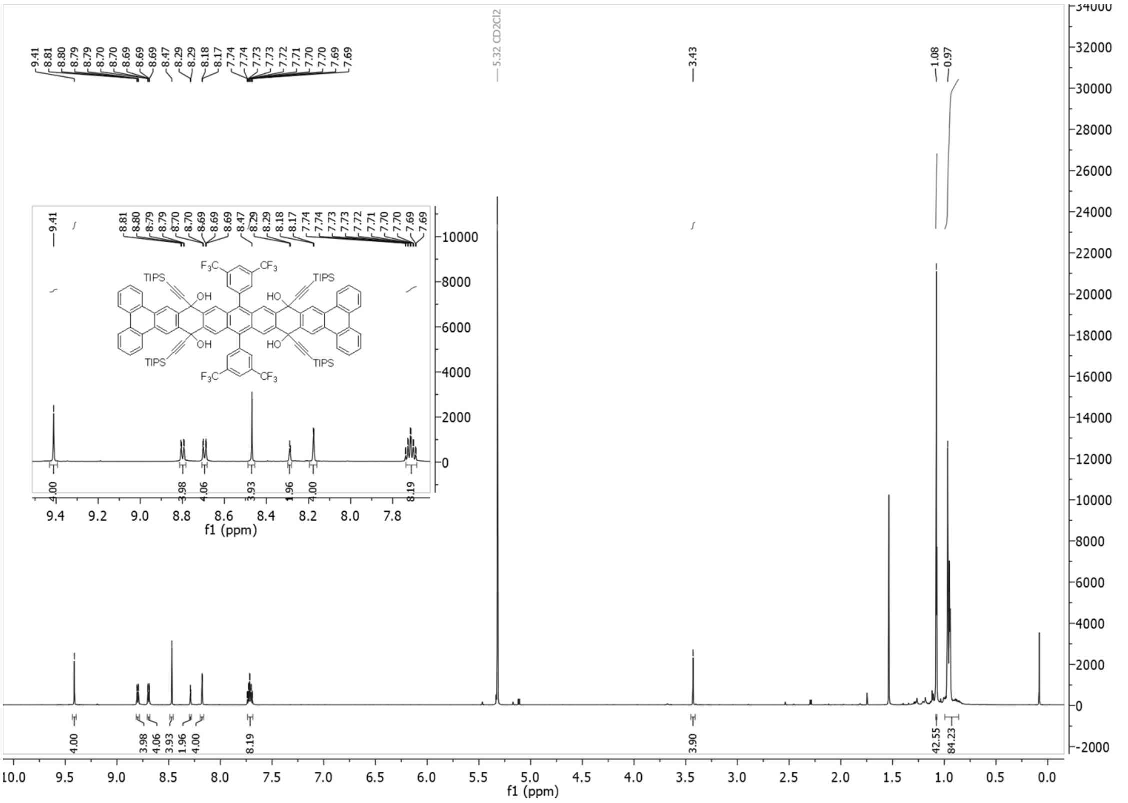

Figure S2. ^{1}H NMR (600 MHz, CD_2Cl_2, 293 K) of **5**.

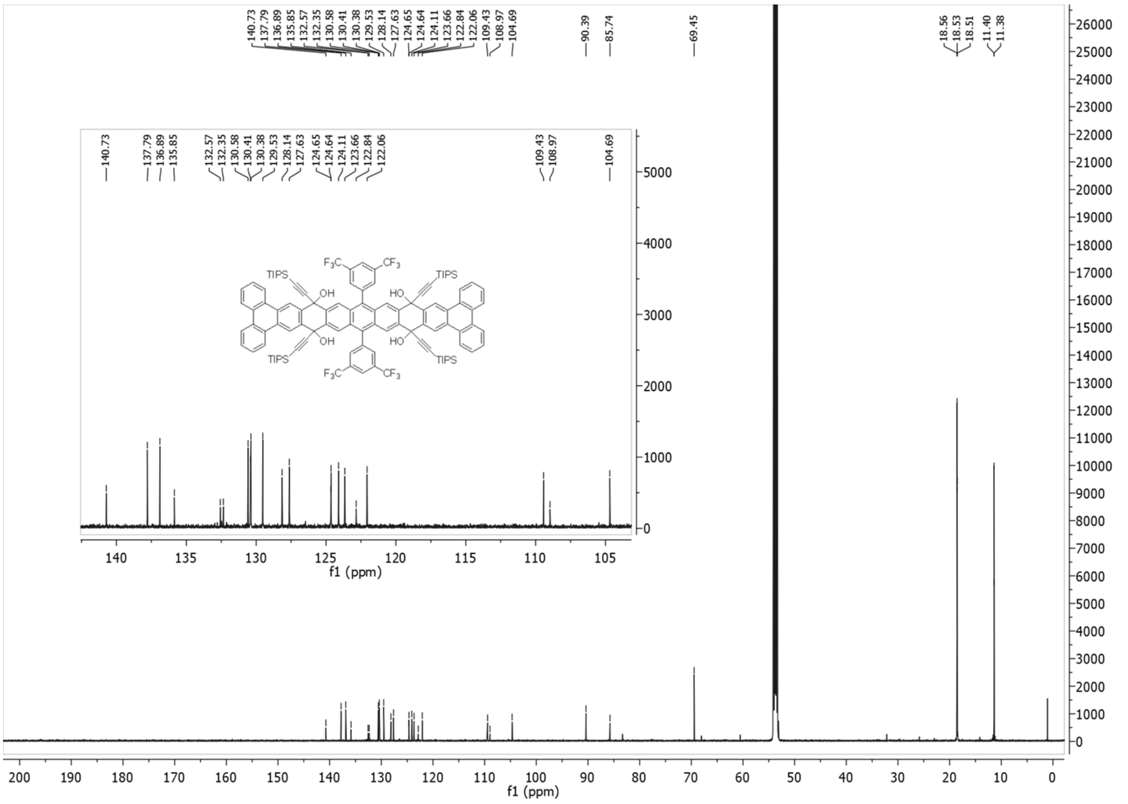

Figure S3. ^{13}C NMR (151 MHz, CD_2Cl_2, 293 K) of **5**.

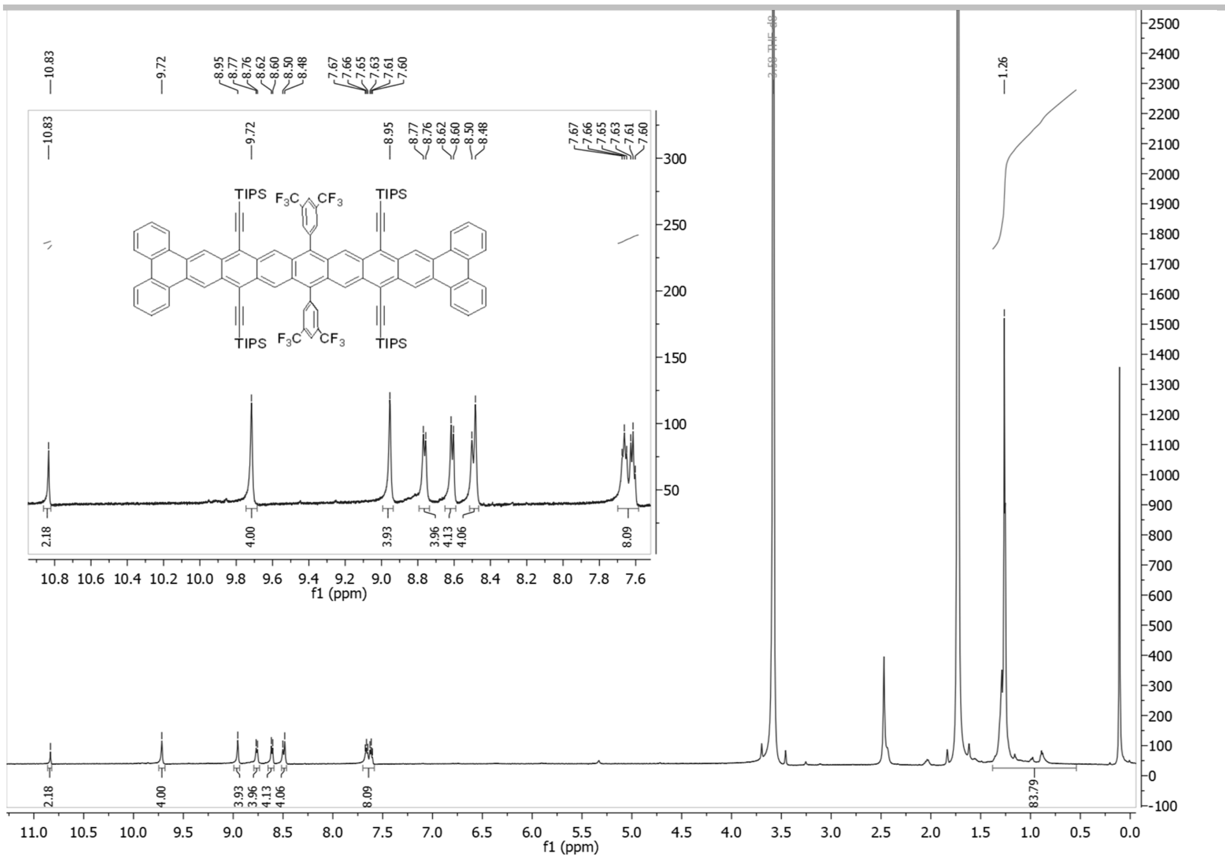

Figure S4. ^{1}H NMR (600 MHz, [D$_8$]THF, 293 K) of **B$_4$Non**.

2.3 IR Spectroscopy

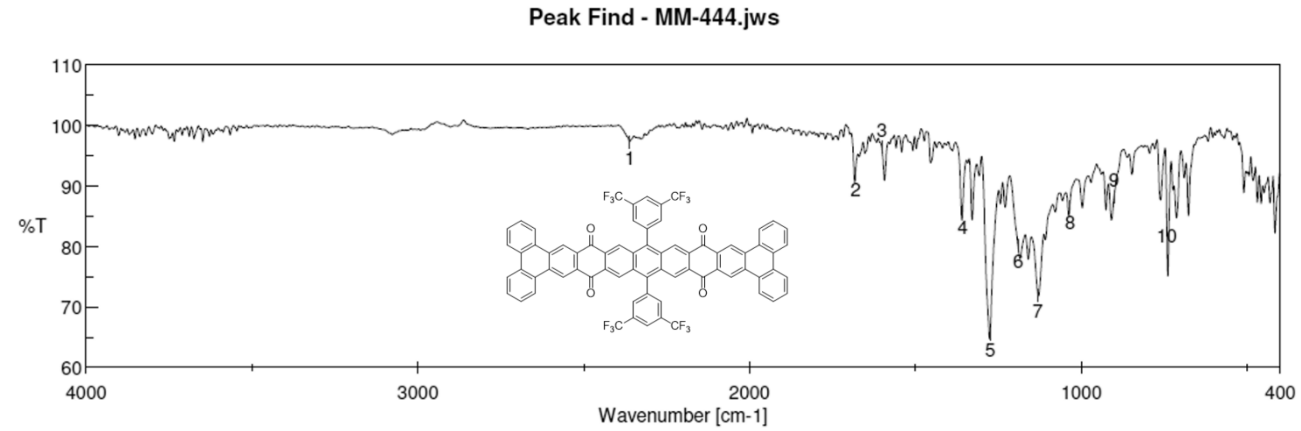

[Result of Peak Picking]

No.	Position	Intensity	No.	Position	Intensity	No.	Position	Intensity
1	2361.41	97.3022	2	1680.66	92.0189	3	1601.59	96.3718
4	1357.64	85.7775	5	1274.72	65.4823	6	1190.83	80.1016
7	1130.08	71.9102	8	1034.62	86.5368	9	901.558	88.1687
10	742.46	84.4406						

Figure S6. IR spectrum of **4** (neat).

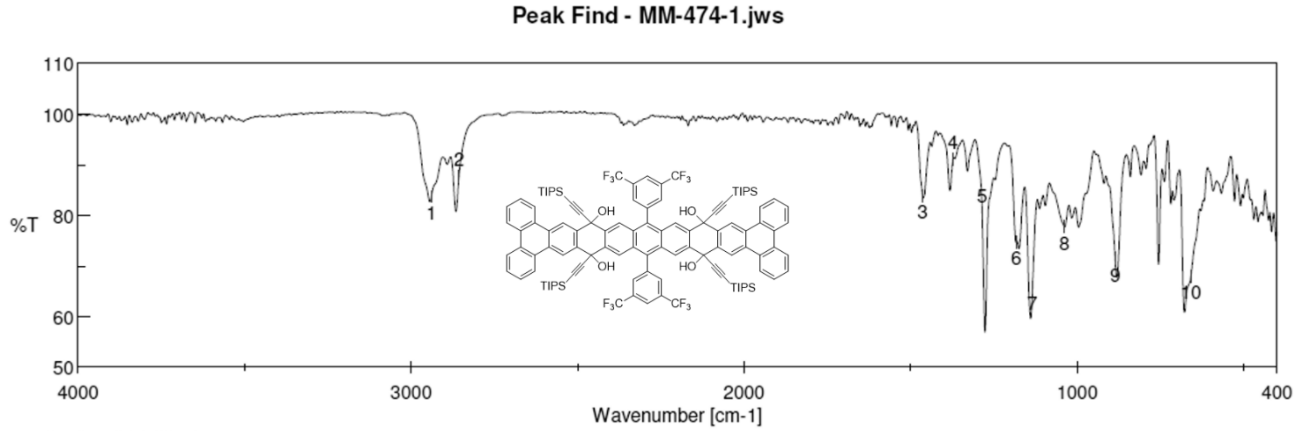

[Result of Peak Picking]

No.	Position	Intensity	No.	Position	Intensity	No.	Position	Intensity
1	2938.98	83.7024	2	2856.06	87.7929	3	1464.67	83.9429
4	1373.07	91.0621	5	1285.32	80.5173	6	1183.11	74.6365
7	1133.94	65.7888	8	1038.48	77.6998	9	886.131	71.4416
10	658.571	67.8739						

Figure S7. IR spectrum of **5** (neat).

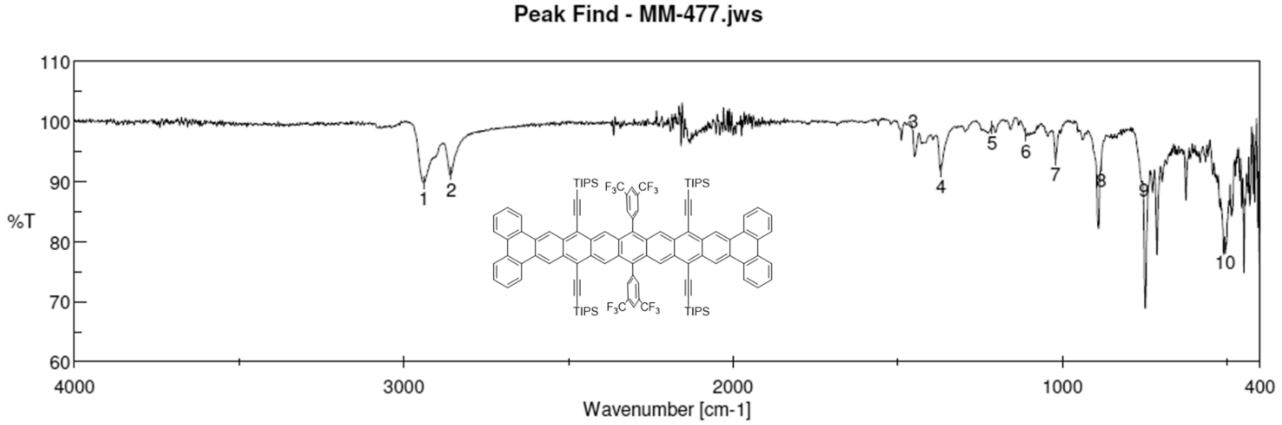

[Result of Peak Picking]

No.	Position	Intensity	No.	Position	Intensity	No.	Position	Intensity
1	2939.47	89.8772	2	2855.58	91.3121	3	1453.1	97.3464
4	1369.21	91.883	5	1213.49	99.1212	6	1110.8	97.7597
7	1019.68	93.8289	8	882.756	92.8916	9	753.548	85.7713
10	506.223	79.1203						

Figure S8. IR spectrum of **B$_4$Non** (neat).

2.4 UV/vis Absorption Stability Study

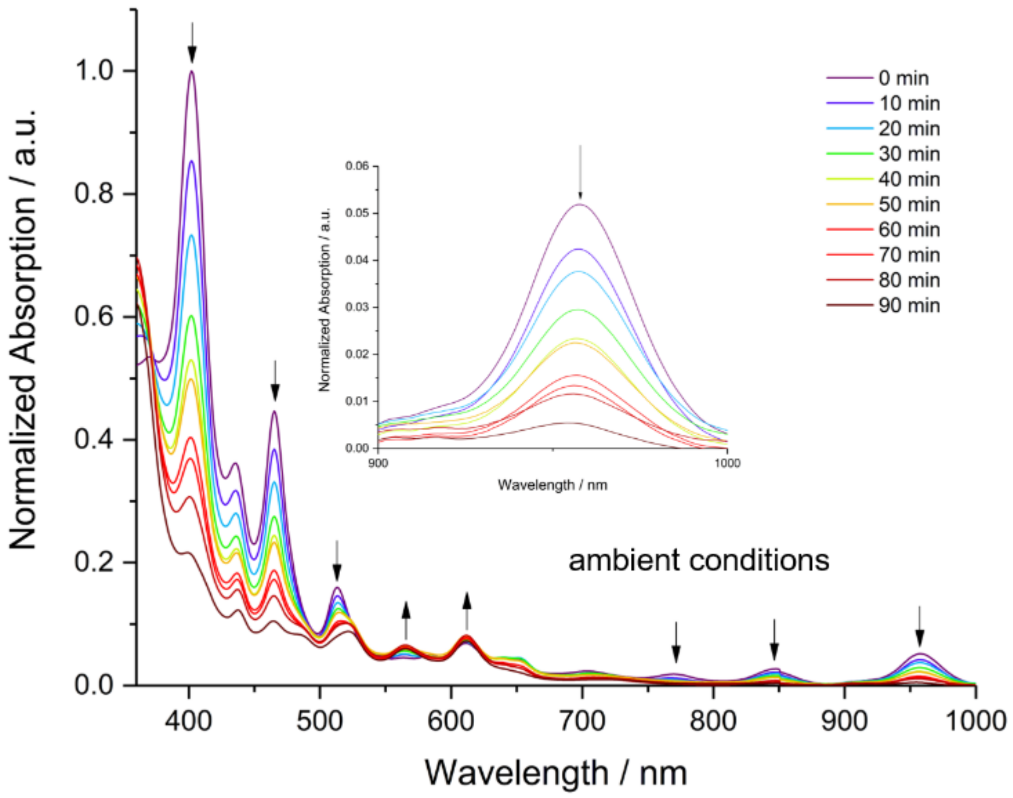

Figure S9. Change in UV/vis absorption intensity of **B_4Non** under ambient conditions in *n*-hexane at room temperature.

2.5 Cyclic Voltammetry

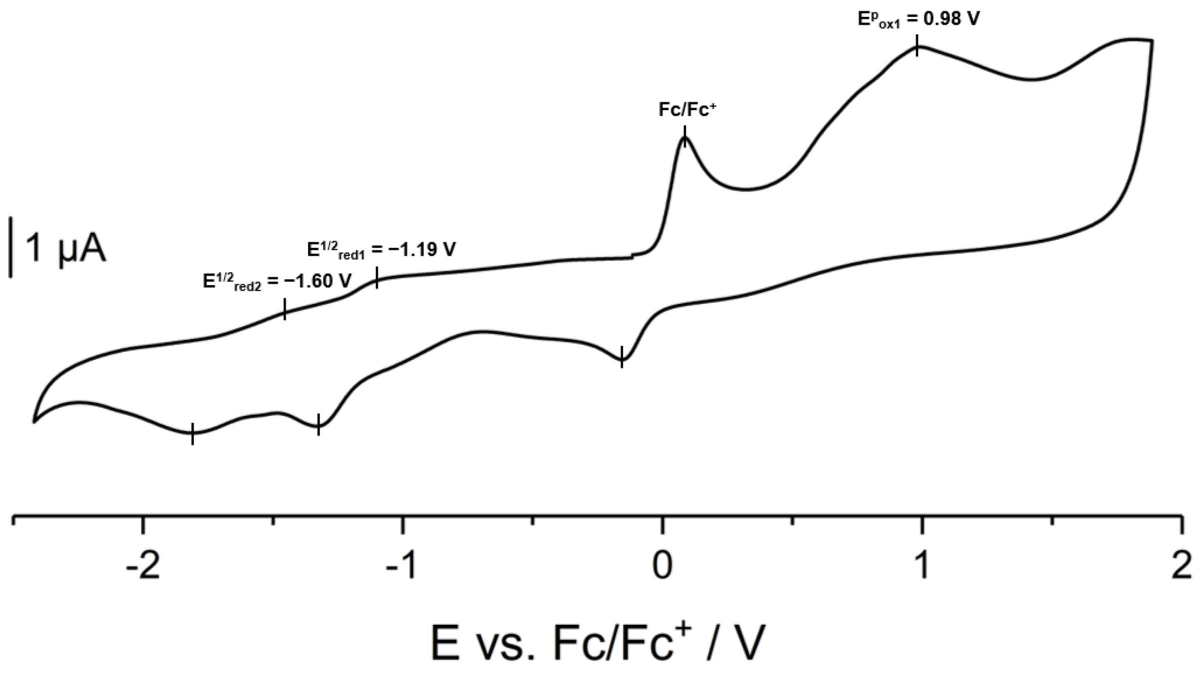

Figure S10. Cyclic voltammogram of **B_4Non** in CH_2Cl_2 using Bu_4NPF_6 as electrolyte, Pt as working electrode, Pt/Ti wire as counter electrode, silver wire as reference electrode and Fc/Fc^+ as internal standard at 0.2 Vs^{-1}.

2.6 Crystallographic Data

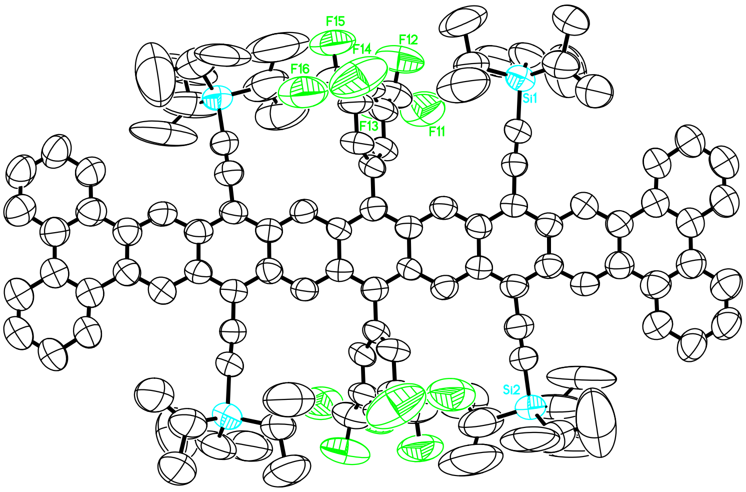

Identification code	1942586	
Empirical formula	$C_{114}H_{114}F_{12}Si_4$	
Formula weight	1824.41	
Temperature	200(2) K	
Wavelength	1.54178 Å	
Crystal system	monoclinic	
Space group	C2/c	
Z	8	
Unit cell dimensions	a = 21.2644(10) Å	□ = 90 deg.
	b = 21.3297(10) Å	□ =99.376(4) deg.
	c = 46.794(2) Å	□ = 90 deg.
Volume	20940.4(17) $Å^3$	
Density (calculated)	1.16 g/cm^3	
Absorption coefficient	1.08 mm^{-1}	
Crystal shape	plank	
Crystal size	0.106 x 0.093 x 0.042 mm^3	
Crystal colour	green	
Theta range for data collection	3.0 to 45.0 deg.	
Index ranges	-8□h□19, -19□k□19, -42□l□42	
Reflections collected	30063	
Independent reflections	8172 (R(int) = 0.0990)	
Observed reflections	5170 (I > 2□(I))	
Absorption correction	Semi-empirical from equivalents	
Max. and min. transmission	1.87 and 0.32	
Refinement method	Full-matrix least-squares on F^2	
Data/restraints/parameters	8172 / 2309 / 1174	
Goodness-of-fit on F^2	2.35	
Final R indices (I>2sigma(I))	R1 = 0.158, wR2 = 0.391	
Largest diff. peak and hole	0.48 and -0.35 $eÅ^{-3}$	

Figure S11. Crystal structure, crystal data and structure refinement of **B_4Non** (CCDC 1942586).

2.7 Analysis of Decomposition Products

Experiment 1:
A freshly prepared solution of **B4Non** in anhydrous THF under inert atmosphere was exposed to ambient conditions, the solvent evaporated and analyzed via MALDI mass spectrometry. Formation of mono-, di- and tri- and even tetra-oxo-adducts is evident (Figure S12):

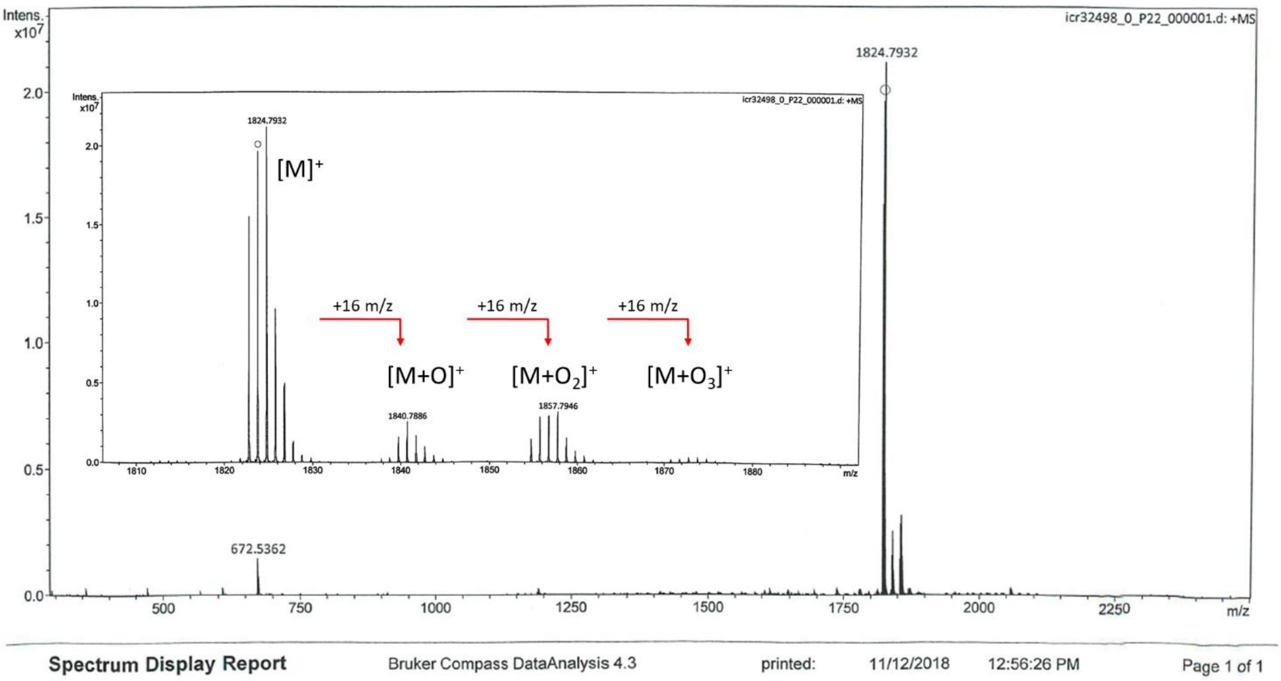

Figure S12. MALDI-MS (DCTB, pos. mode) of the product obtained from degradation under ambient conditions showing oxo-adducts.

Further structure elucidation via NMR or single crystal analysis was not met with success: out of the mixture, we could not pinpoint the exact location(s) of oxidation and could not obtain single crystals of these oxo-adducts suitable for crystallographic analysis.

Experiment 2:
A solution of **B4Non** in anhydrous THF was left standing under nitrogen in the dark for several days. Chromatographic separation was carried out using preparative recycling gel permeation chromatography, its trace is depicted in Figure S13. A complex mixture of decomposition products is formed. Note that despite one peak looking prominent, this was not reflected with regard to the actual mass isolated, so we presume its abundance is overestimated. The major product peak was isolated in fraction one with is mass spectrum (MALDI, DCTB, pos. mode) depicted in Figure S14 and its absorption spectrum in $CHCl_3$ depicted in Figure S15 (black line) – the other byproducts were collected in total as a second fraction (Figure S15 red line, Figure S16). The absence of the starting material is evident from all spectra. Mass spectrometry of the main decomposition product suggests a dimeric species as an adduct with another fragment of unknown identity and chromophores no longer than substituted anthracene cores. The minor fraction probably contains even higher oligomeric species – its absorption spectrum in $CHCl_3$ also supports the presence of anthracene-like chromophors and, to some extent, traces of oxygenated species resulting from non-ideal inert conditions during our decomposition experiment. NMR analysis of both fractions (Figure S17, S18) suggests a mixture of multiple compounds being present. Resonances between 5.0 and 5.8 ppm[S5] support both the presence of bridge head atoms resulting from dimerization/oligomerization (via formal [4+4] or Diels-Alder reaction).

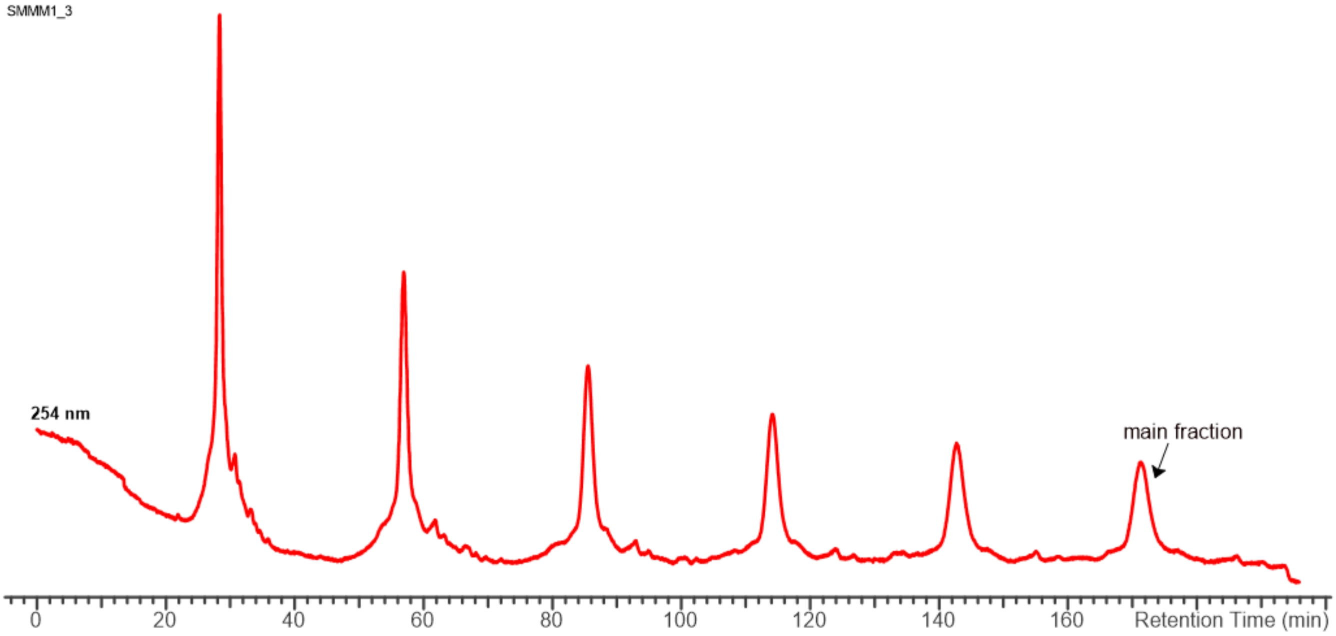

Figure S13. Recycling GPC trace of **B₄Non** after allowing it to degrade for several days in THF solution under nitrogen in the dark.

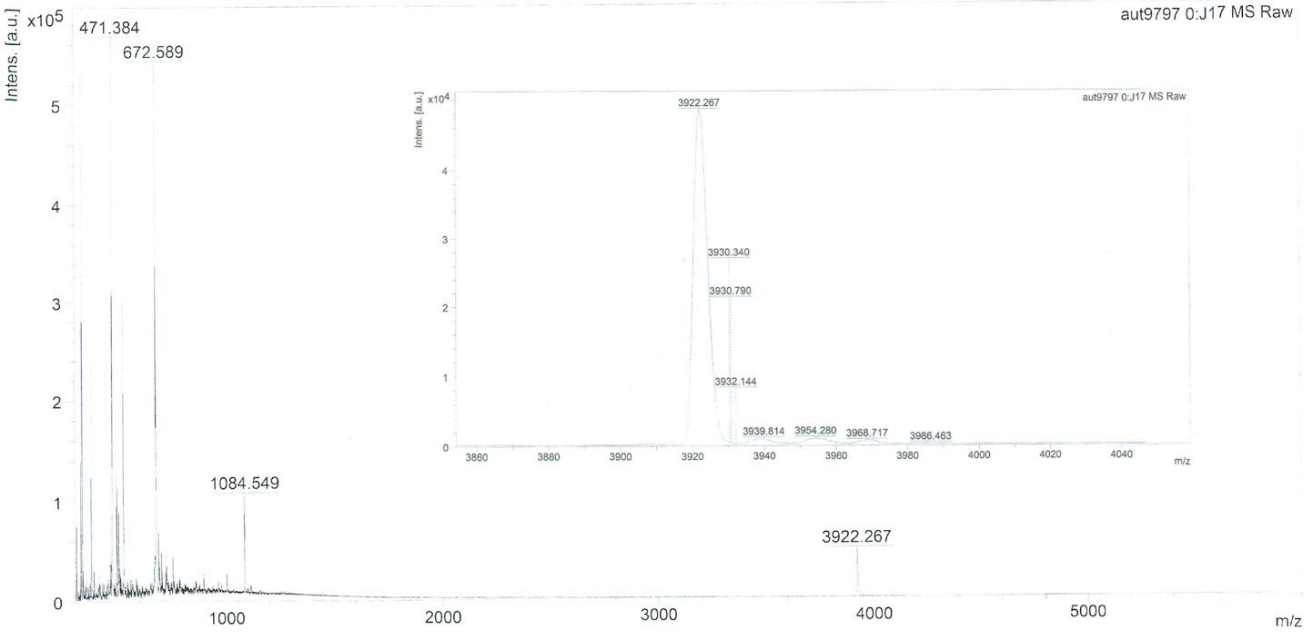

Figure S14. MALDI-MS (DCTB, pos. mode) of fraction one showing higher m/z than **B₄Non** (m/z = 1823). Further structure elucidation via NMR or X-ray single crystal analysis was not met with success.

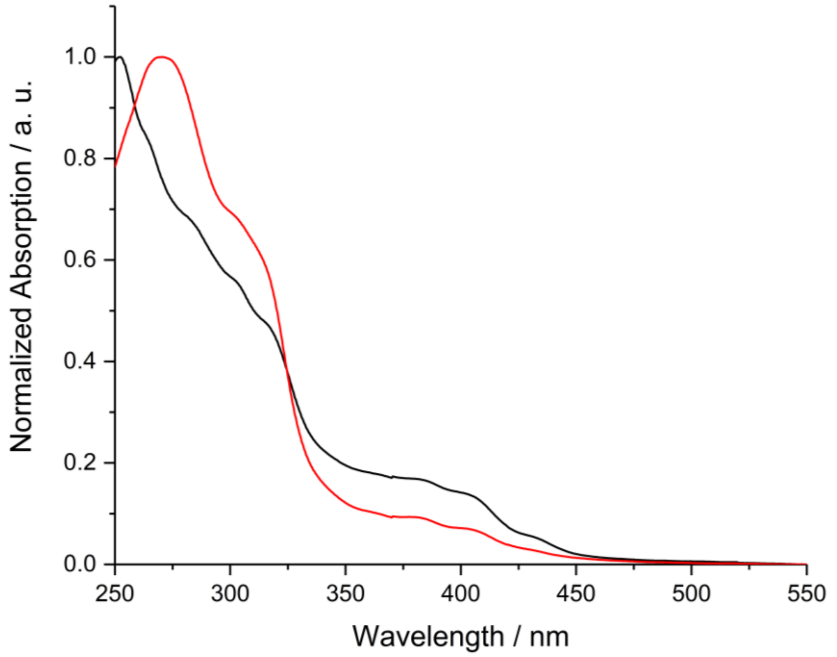

Figure S15. UV/vis absorption spectrum in $CHCl_3$ at room temperature of the main degradation product (fraction 1: black; fraction 2: red).

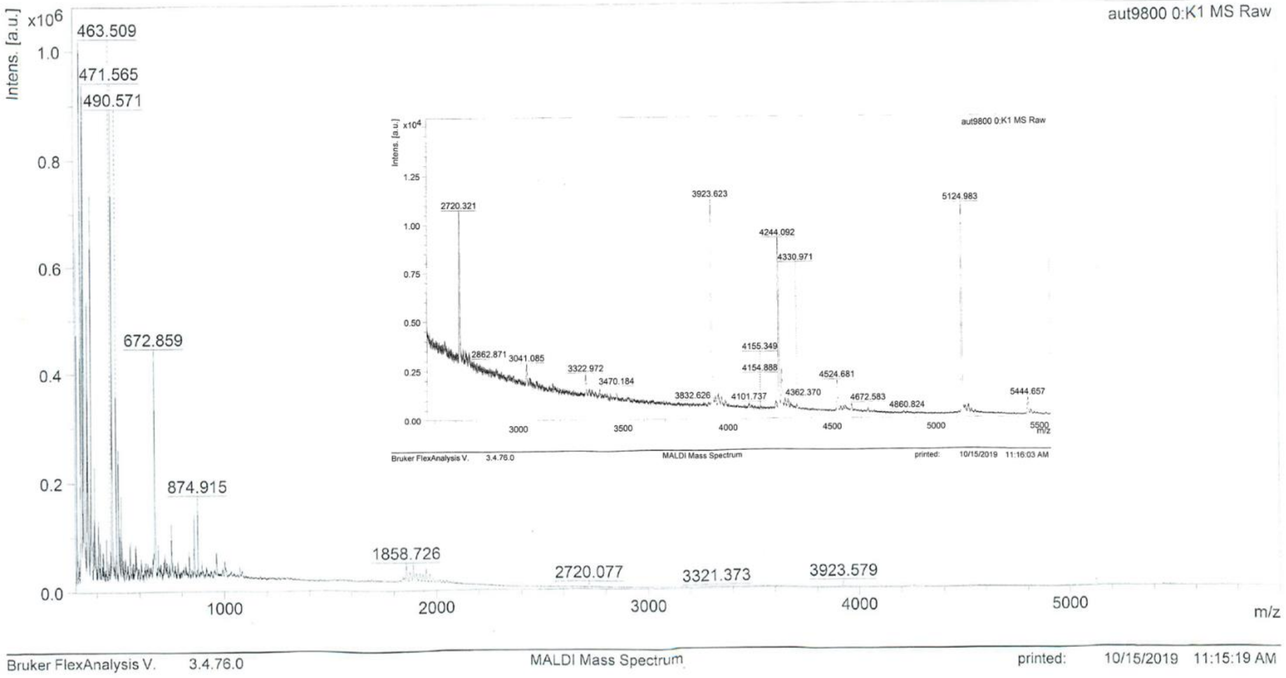

Figure S16. MALDI-MS (DCTB, pos. mode) of faction 2 showing higher m/z than B_4Non (m/z = 1823). Further structure elucidation via NMR or X-ray single crystal analysis was not met with success.

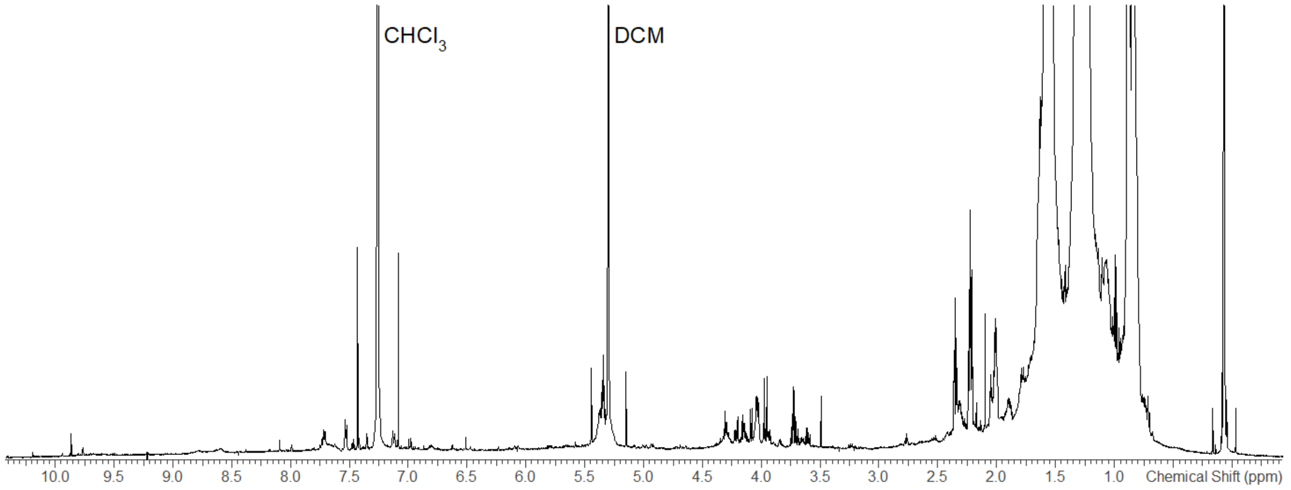

Figure S17. Magnified ^{1}H NMR (600 MHz, $CDCl_3$, rt) of fraction 1 illustrating various decomposition products. Solvent signals: $CHCl_3$ and DCM in addition to their satellite signals.

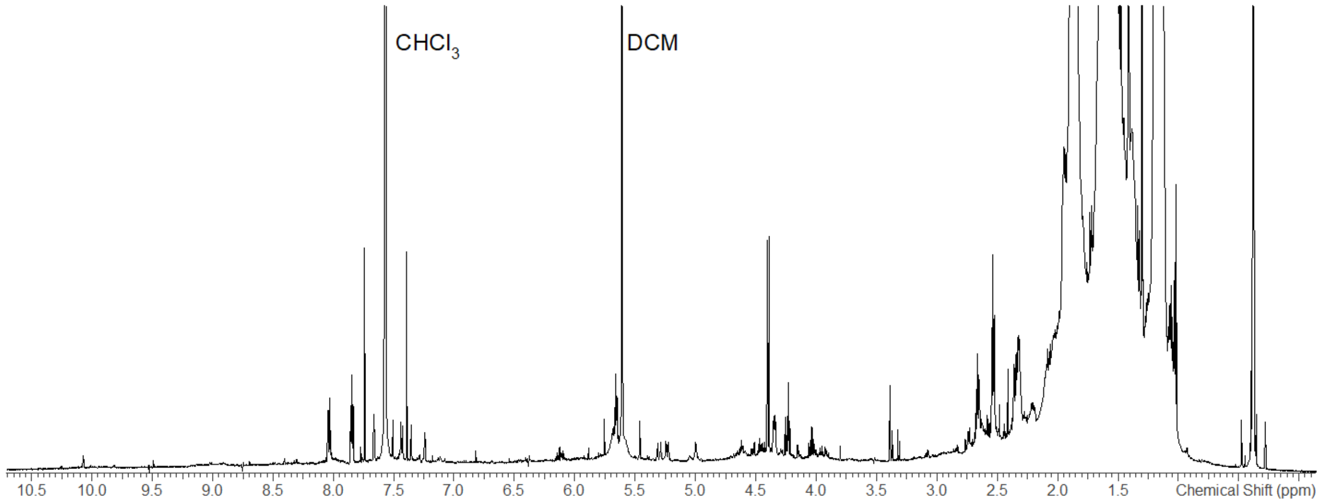

Figure S18. Magnified ^{1}H NMR (600 MHz, $CDCl_3$, rt) of fraction 2 illustrating various decomposition products. Solvent signals: $CHCl_3$ and DCM in addition to their satellite signals.

References

[S1] H. E. Gottlieb, V. Kotlyar, A. Nudelman, *J. Org. Chem.* **1997**, *62*, 7512-7515.

[S2] G. W. T. M. J. Frisch, H. B. Schlegel, G. E. Scuseria, M. A. Robb, J. R. Cheeseman, I. G. Scalmani, V. Barone, B. Mennucci, G. A. Petersson, H. Nakatsuji, M. Caricato, X. Li, H. P. Hratchian, A. F. Izmaylov, J. Bloino, G. Zheng, J. L. Sonnenberg, M. Hada, M. Ehara, K. Toyota, R. Fukuda, J. Ha- segawa, M. Ishida, T. Nakajima, Y. Honda, O. Kitao, H. Nakai, T. Vreven, J. A., Jr., Montgomery, J. E. Peralta, F. Ogliaro, M. J. Bearpark, J. Heyd, E. N. Brothers, K. Kudin, V. N. Staroverov, R. Kobayashi, J. Normand, K. Raghavachari, A. P. Rendell, J. C. Burant, S. S. Iyengar, J. Tomasi, M. Cossi, N. Rega, N. J. Millam, M. Klene, J. E. Knox, J. B. Cross, V. Bakken, C. Adamo, J. Jaramillo, R. Gomperts, R. E. Stratmann, O. Yazyev, A. J. Austin, R. Cammi, C. Pomelli, J. W. Ochterski, R. L. Martin, K. Morokuma, V. G. Zakrzewski, G. A. Voth, P. Salvador, J. J. Dannenberg, S. Dapprich, A. D. Daniels, O. Farkas, J. B. Foresman, J. V. Ortiz, D. J. Cioslowski, D. Fox, J., *Gaussian09*, Wallingford, **2009**.

[S3] R. P. Kreher, T. Hildebrand, *Chem. Ber.* **1988**, *121*, 81-87.

[S4] B. Purushothaman, M. Bruzek, S. R. Parkin, A.-F. Miller, J. E. Anthony, *Angew. Chem. Int. Ed.* **2011**, *50*, 7013-7017.

[S5] B. Purushothaman, S. R. Parkin, J. E. Anthony, *Org. Lett.* **2010**, *12*, 2060–2063.

Author Contributions

Matthias Müller: Synthesis, analysis, writing of original draft (lead)
Steffen Maier: Synthesis, cyclic voltammetry measurements (supporting)
Olena Tverskoy: Synthesis of precursors (supporting)
Frank Rominger: Crystal structure elucidation (supporting)
Jan Freudenberg: Project administration, editorial work (supporting)
Uwe H. F. Bunz: Writing of original draft, project administration, funding acquisition (lead)
